# Efficacy of Lateral Sacroiliac Joint Fusion With the Trident™ Screw System: A Retrospective Analysis

**DOI:** 10.7759/cureus.77793

**Published:** 2025-01-21

**Authors:** Hamid Abbasi, Dominic Moore, Mitch A Rusten, Jiawen Zhan, Adam Sima, Twanesha Lightbourn

**Affiliations:** 1 Spine Surgery, Avicenna Technical University and Inspired Spine Health, Burnsville, USA; 2 Spine Surgery, Inspired Spine Health, Burnsville, USA; 3 Research, Inspired Spine Health, Burnsville, USA; 4 Applied AI and Programming, Avicenna Technical University, Burnsville, USA; 5 Clinical Medicine, Nura Pain Clinics, Burnsville, USA; 6 Clinical Medicine, Inspired Spine Health, Burnsville, USA

**Keywords:** chronic low back pain (clbp), minimally invasive spine surgery, sacroiliac fusion, sacroiliac joint, sacroiliac joint fixation

## Abstract

Background

Chronic lower back pain is a significant global health issue, leading to disability and a substantial economic burden. A considerable number of cases are associated with the sacroiliac joint (SIJ), especially among patients with a history of lumbar fusions. Despite various treatments, persistent SIJ pain often requires surgical intervention, with minimally invasive techniques becoming the standard due to their benefits over open surgery. This study examines the clinical outcomes of a novel minimally invasive SIJ fixation technique using the Trident™ system.

Methodology

The study retrospectively reviewed 39 patients who underwent SIJ fusion with the Trident™ system across four surgical sites by a single surgeon. Strict inclusion and exclusion criteria ensured a homogeneous patient population. The procedure involved placing hollow fenestrated screws in a tri-pronged fashion across the SIJ to achieve fusion. The Oswestry Disability Index (ODI) was used to measure functional outcomes, and statistical analyses were conducted to assess the efficacy of the procedure.

Results

The study found statistically significant improvements in most ODI categories postoperatively. Subgroup analyses revealed that patients with bilateral SIJ fusions showed the greatest improvement, while those with unilateral fusions without prior lumbar surgery showed the least improvement, though still significant. The results suggest that the Trident™ system is effective in improving patient outcomes.

Conclusions

Evidence indicates that the novel SIJ fixation technique could influence future standards of care for SIJ-related chronic low back pain. The Trident™ Screw System’s minimally invasive approach to SIJ fusion is effective in improving functional outcomes for patients with chronic SIJ-related lower back pain. The technique’s efficacy, demonstrated through significant ODI improvements, supports its potential as a valuable treatment option.

## Introduction

Chronic lower back pain is a significant medical challenge, affecting approximately 9.4% of the global population and serving as the leading contributor to disability worldwide [1]. The economic burden of this condition is substantial, with spine-related healthcare costs showing an upward trend, exemplified by the $85.9 billion expenditure recorded in 2005 [2]. Lower back pain can originate from various anatomical sources, with a notable proportion of cases linked to the lumbar intervertebral discs, facet joints, and the sacroiliac joint (SIJ) [3]. Notably, the SIJ is implicated in up to 30% of chronic low back pain presentations and assumes a greater prevalence among patients with a history of lumbar fusions [4].

The SIJ, innervated by lumbosacral nerve roots, is designed for limited physiological movement, secured by robust ligaments. The etiology of SIJ pain is not entirely elucidated but is hypothesized to stem from abnormal joint motion, leading to inflammation and subsequent pain [5]. Current therapeutic strategies for SIJ pain encompass nociceptive nerve ablation, anti-inflammatory interventions, and, as a definitive solution, joint fusion. Conservative treatments such as physical therapy, therapeutic sacroiliac injections, and radiofrequency ablation serve as initial modalities, although their long-term efficacy remains a subject of debate [6,7]. Persistent SIJ pain unresponsive to conservative measures often necessitates permanent intervention in the form of SIJ fusion.

Historically, SIJ fusion entailed an open surgical approach, requiring a sizable incision and extensive joint preparation [8]. However, the evolution of surgical techniques has ushered in an era of minimally invasive procedures, which have become the preferred method due to their reduced tissue damage, lower blood loss, shorter operative times, and expedited recovery. By 2012, minimally invasive techniques accounted for 85% of SIJ fusions [9]. Despite this progress, there is no unified agreement on the optimal minimally invasive technique, with various methods currently in practice [8]. One such technique involves the percutaneous implantation of triangular titanium implants to obtain transarticular stabilization and long-term biological fixation of the joint via fusion [10].

This article focuses on the clinical outcomes following a novel approach to SIJ fixation, utilizing a lateral incision to place hollow fenestrated screws in a tri-pronged fashion across the joint. This study aims to provide valuable insights into the efficacy of this innovative technique, potentially influencing future standards of care for patients suffering from SIJ-related chronic low back pain with measurable metrics of pain reduction and improved functionality.

## Materials and methods

This study was designed as a retrospective chart review focusing on SIJ fusion procedures conducted using the Trident™ system. The setting for these procedures encompassed four surgical sites, but following a single surgeon to isolate any discrepancies in technique. The locations included Inspired Spine Surgical Center in Burnsville, MN; River View Health in Crookston, MN; Summit Surgical in Hutchinson, KS; and Maple Grove Surgery Center in Maple Grove, MN. Ethical approval for the study was granted by Pearl Pathways (approval number: #21-INSS-102) following the guidelines of FDA 21 CFR 56.104 and 45CFR46.104(b).

There were 39 patients in total who qualified for inclusion in this study. The inclusion criteria for the study were strictly defined to ensure a homogeneous patient population. Participants were required to be 18 years or older, having undergone SIJ fusion procedures performed by the surgeon from January 1, 2021, to February 1, 2024, and having at least six months of chart data available. They needed to meet clinical diagnostic criteria for SIJ fusion, including a characteristic history and positive results on at least three out of five provocation tests, positive diagnostic injections, and a history of failed conservative therapy. Exclusion criteria were established to omit patients with factors that could confound the study results, such as participation in investigational programs, pregnancy, use of SIJ fusion devices other than Trident, age under 18 years, documented psychological or social disorders, possible financial gain, and life-threatening or terminal medical conditions.

**Figure 1:**
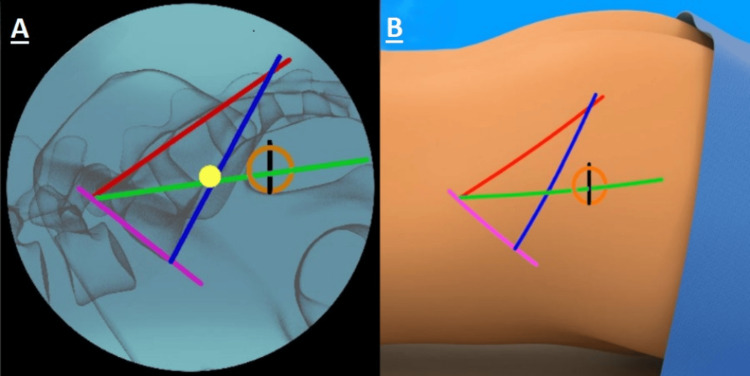
The visualization used for marking, with imaging of the fluoroscopy screen on left (A), and how it corresponds to the patient as true visual on the right (B). Lateral fluoroscopy was used to visualize the greater sciatic notch, sacral endplate (pink line), and ala. The skin was marked at the shadow of the sacrum (red and blue lines) using a guidewire and marking pen. The mark was made from the posterior edge of the sacral endplate to the middle of the sciatic notch (green line). The tip of the guidewire was pulled back to the middle of the sciatic notch along the green line, and a perpendicular mark (black line) was drawn to 1 inch above this location.

The surgical procedure used with the Trident system followed a meticulous protocol to ensure consistency across all cases. After undergoing general anesthesia, or a twilight form of sedation known as monitored anesthesia care, patients were placed in a prone position with the affected side marked and identified. C-arm fluoroscopy was utilized for intraoperative imaging, and electromyography or equivalent neuromonitoring was employed to monitor muscle activity, enhancing procedural safety. For the use of C-arm fluoroscopy in site preparation, see Figure 1.

After set up, an incision was made along the 1-inch vertical line. The guidewire was inserted from lateral to medial through the incision, and the blunt tip of the guidewire was placed 1.5-2 inches above the ala and perpendicular to the SIJ. When the guidewire was in this approximate location, the 8 mm dilator was delivered over the guidewire up to the surface of the ilium. The guidewire was removed, flipped, and re-inserted with the trocar tip against the ilium. The guidewire was tapped slightly to advance a few millimeters into the cortical surface, and the C-arm was adjusted from lateral to inlet and outlet views to confirm the correct trajectory.

**Figure 2:**
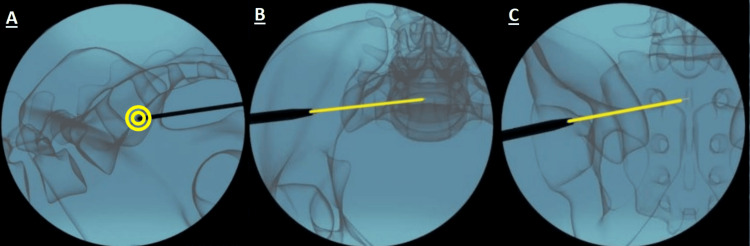
Fluoroscopic image replicas of guidewire placement with (labeled from left to right) (A) lateral, (B) inlet, and (C) outlet views represented. The yellow circle in the lateral view shows the initial placement of the blunt tip of the guidewire. The following yellow lines show the trajectory.

Once the correct iliac contact point and approach angle were confirmed, the guidewire was advanced past the sacroiliac joint using a drill. The usual target (in the outlet view of Figure 2) was just inferior to the S1 foramen. The trajectory placed the guidewire into the center of mass of the S1-S2-S3 segment of the sacrum.

**Figure 3:**
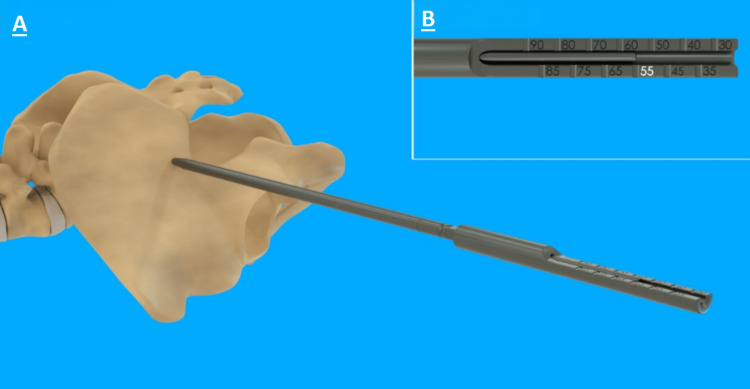
Image of guidewire with the 8 mm dilator and measuring sleeve to show the implant length of 55 mm. Labeled on the left (A) is the operative view, showing how the instrument interacts with the patient anatomy, and a more detailed image of the device labeled (B) on the top right.

When the guidewire was at the full depth intended, the measuring sleeve was used with the 8 mm dilator to confirm the preoperative Ø13 mm main screw length selection. The measuring sleeve indicated the distance of the guidewire past the tip of the 8 mm dilator, as shown in Figure 3.

The 22 mm dilator was placed over the 8 mm dilator. The 8 mm dilator was removed while leaving the guidewire in place. The 8 mm cannulated drill was then used to create a small cavity in the cortical surface of the ilium, without extending more than a few millimeters into the cancellous bone. This preparation allowed the tip of the 13 mm Trident screw to easily enter the space so that the self-tapping flutes could engage the cortical bone. This surgical process does not require repeat instrument placement and drilling like some other robust systems [11]. The 8 mm cannulated drill was removed, leaving the guidewire in place. Finally, the 22 mm dilator was removed.

The implants were selected based on the measuring sleeve and preoperative measurements. The same length of the implant was sometimes appropriate for the Ø13 mm main screw and the Ø6 mm side screw. This was determined using fluoroscopic imaging and pre-surgical measuring of imaging after the Ø13 mm main screw was in place. A Trident Ø13 mm main screw was assembled onto the introducer sleeve by matching the protruding tabs of the sleeve to the corresponding spaces in the screw head and twisting clockwise. The square driver was placed into the cannulation of the introducer sleeve, and the preferred handle was attached to the proximal end of the square driver. The multifunctional handle was used axially or as a T-handle. Corresponding cutouts on one end and in the middle of the multifunctional handle allowed it to be used in both orientations.

The 13 mm main screw, square driver, and introducer sleeve were inserted over the guidewire. When the tip of the Trident Ø13 mm main screw contacted bone, screwing into the ilium was initiated.

**Figure 4:**
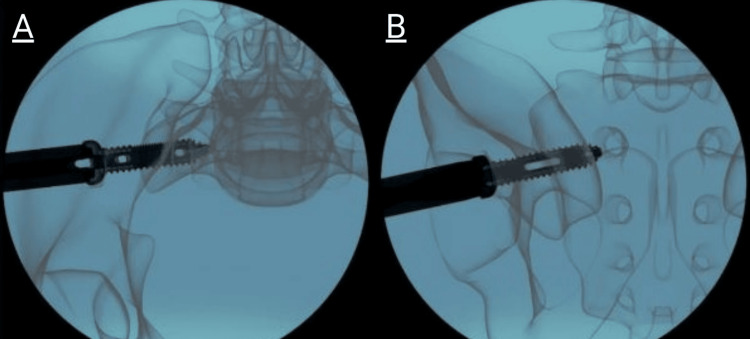
Image of Trident Ø13 mm main screw insertion in appropriate alignment in both inlet (A) and outlet (B) views.

Neuromonitoring was checked during the insertion of the Ø13 mm main screw. The Ø13 mm main screw was advanced until the flange contacted the ilium. The inlet view of the pelvis was used to align the Trident Ø13 mm main screw, ensuring proper orientation for the Trident Ø6 mm side screws. After the flange was in contact with the cortical surface of the ilium, rotation was continued until the two small windows of the Trident Ø13 mm main screw were aligned and overlapped in the inlet view, as shown in Figure 4. The skin markings for the anterior and posterior walls of the sacrum were used as a second reference to confirm that the orientation of the Ø6 mm side screw trajectory was within the sacrum.

**Figure 5:**
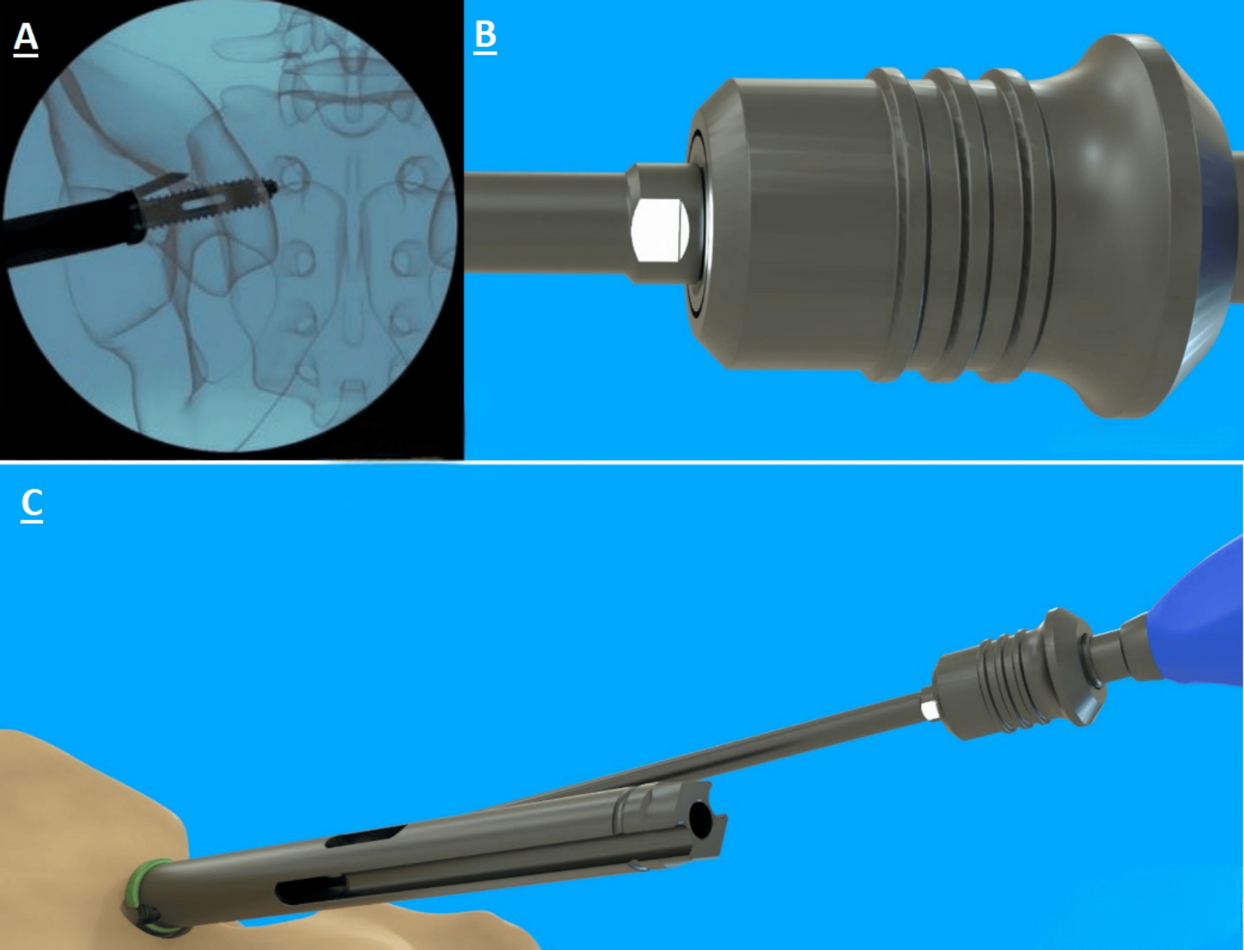
Orientation of awl for Ø6 mm side screw preparation. Outlet view (A) shows how it appears on imaging. The connection (B) is shown with the flat facing in white. This white flat is also shown in use of the instrument (C).

The handle, square driver, and guidewire were removed, but the introducer sleeve was left in place. Care was taken to ensure that the introducer sleeve did not rotate after the square driver was removed. The awl was connected to the quick-connect palm handle. The orientation of the awl tip was noted, along with its correlation to the alignment flat near the quick-connect handle. Figure 5 provides a visual example. The awl was placed through the introducer sleeve, and a mallet was used to advance up to 25 mm into the cortical surface of the ilium to prepare a pathway for the Ø6 mm side screw. This process was repeated for the opposite side.

The side screw driver was connected to the appropriate length Ø6 mm side screw. The threaded tip of the side screw driver was advanced by turning clockwise until the hexalobe was engaged.

**Figure 6:**
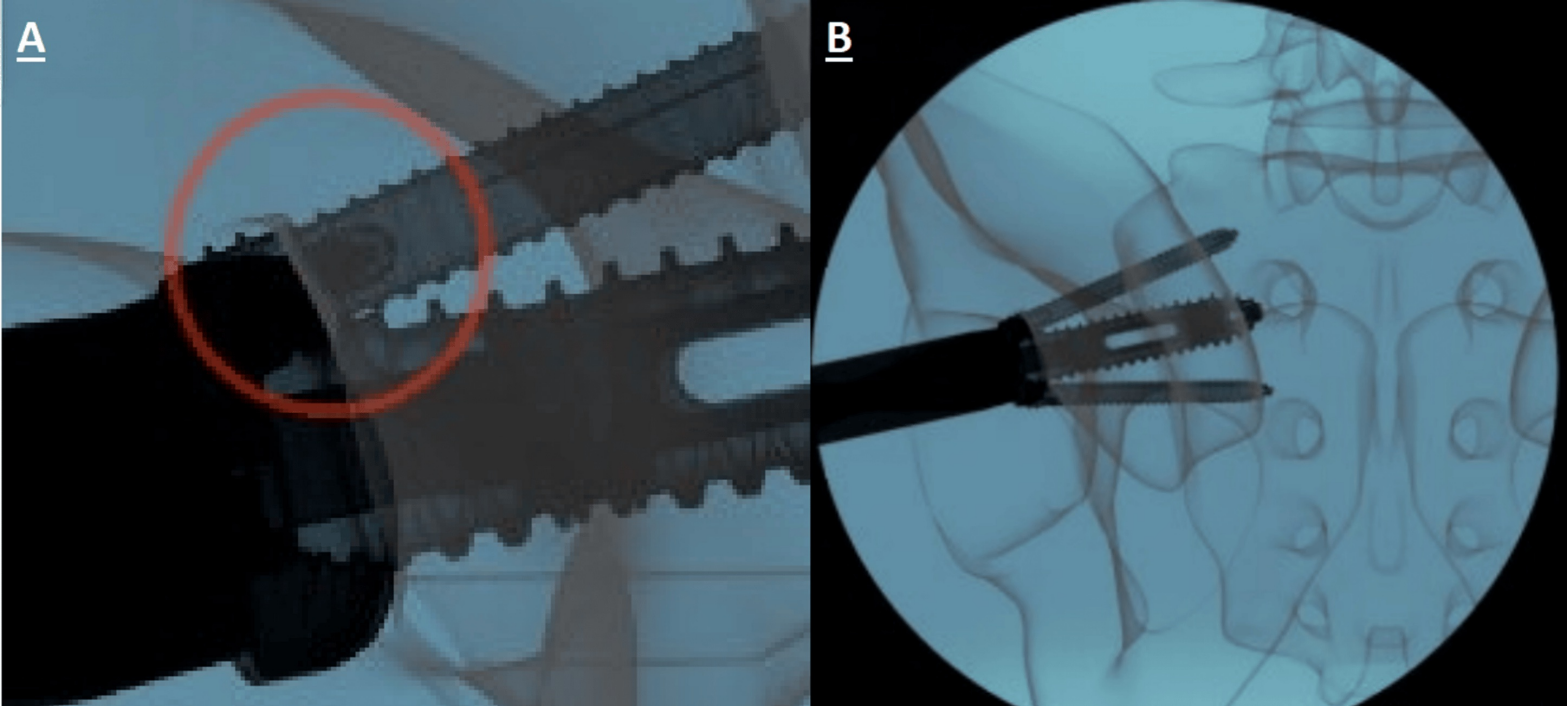
Ø6 mm side screws in position. The head of the side screw, as visualized under fluoroscopy, is shown in the left image (A), while the right (B) displays the final outlet view.

As the Ø6 mm side screw approached its final location, the cavity inside the Ø6 mm side screw head became visible on fluoro images, as seen in Figure 6. The side screw driver was rotated until the Ø6 mm side screw stopped within the Ø13 mm main screw. The final half-turn of the Ø6 mm side screw was preceded by slight resistance as the threads reached an interference fit. After this interference, a half-turn was completed to place the Ø6 mm side screw in its final location and set the anti-backout feature.

The side screw driver was removed by pulling it back 3 mm to disengage the hexalobe drive and then rotating counterclockwise to unthread the retainer tip. The Ø6 mm side screw delivery steps were repeated for the opposing Ø6 mm side screw. The placement of the Trident Ø6 mm side screws was confirmed on the outlet view of fluoroscopy, as shown in Figure 6.

The graft delivery sleeve was placed into the introducer sleeve. The graft tamp was used to place bone graft materials, a mixture of tricalcium phosphate and bone marrow, into the core of the Trident Ø13 mm main screw to promote osseous bridging. The graft tamp and graft delivery sleeve were removed by pulling them out of the introducer sleeve.

**Figure 7:**
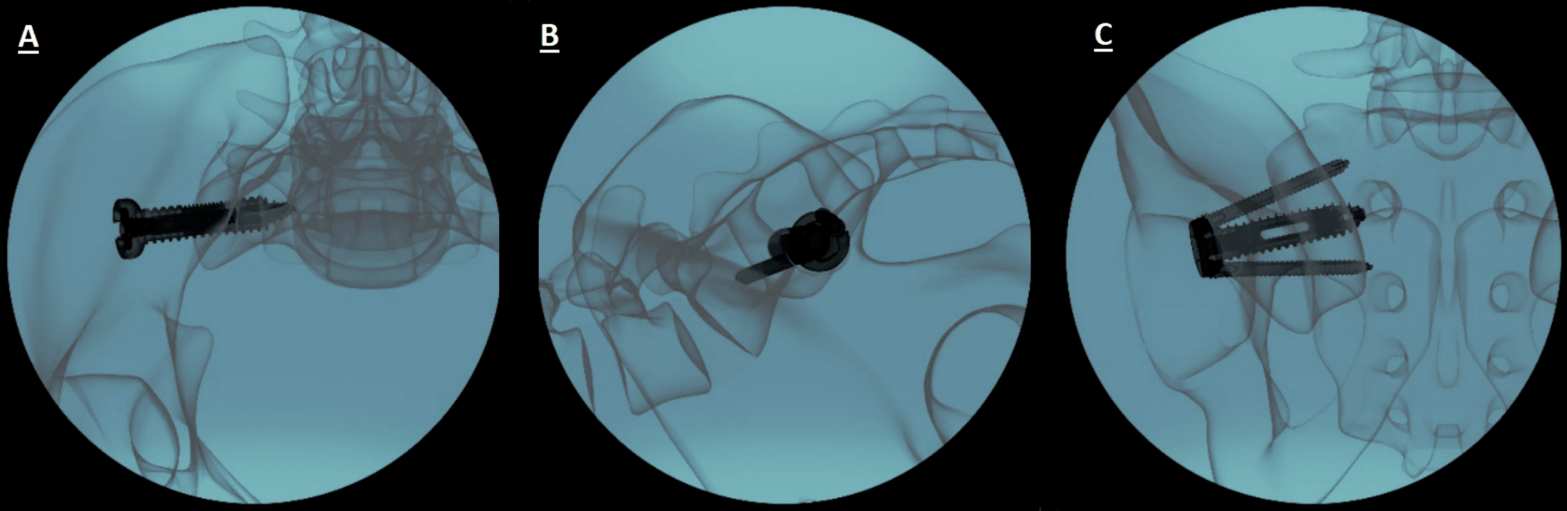
The full Trident system engaged across the sacroiliac joint in three perspectives (labeled from left to right) as (A), (B), and (C) for inlet, lateral, and outlet views, respectively. It can be seen how the secondary screws are positioned inferiorly and superiorly to deny any rotation around the main screw and assure immediate fixation of the joint.

**Figure 8:**
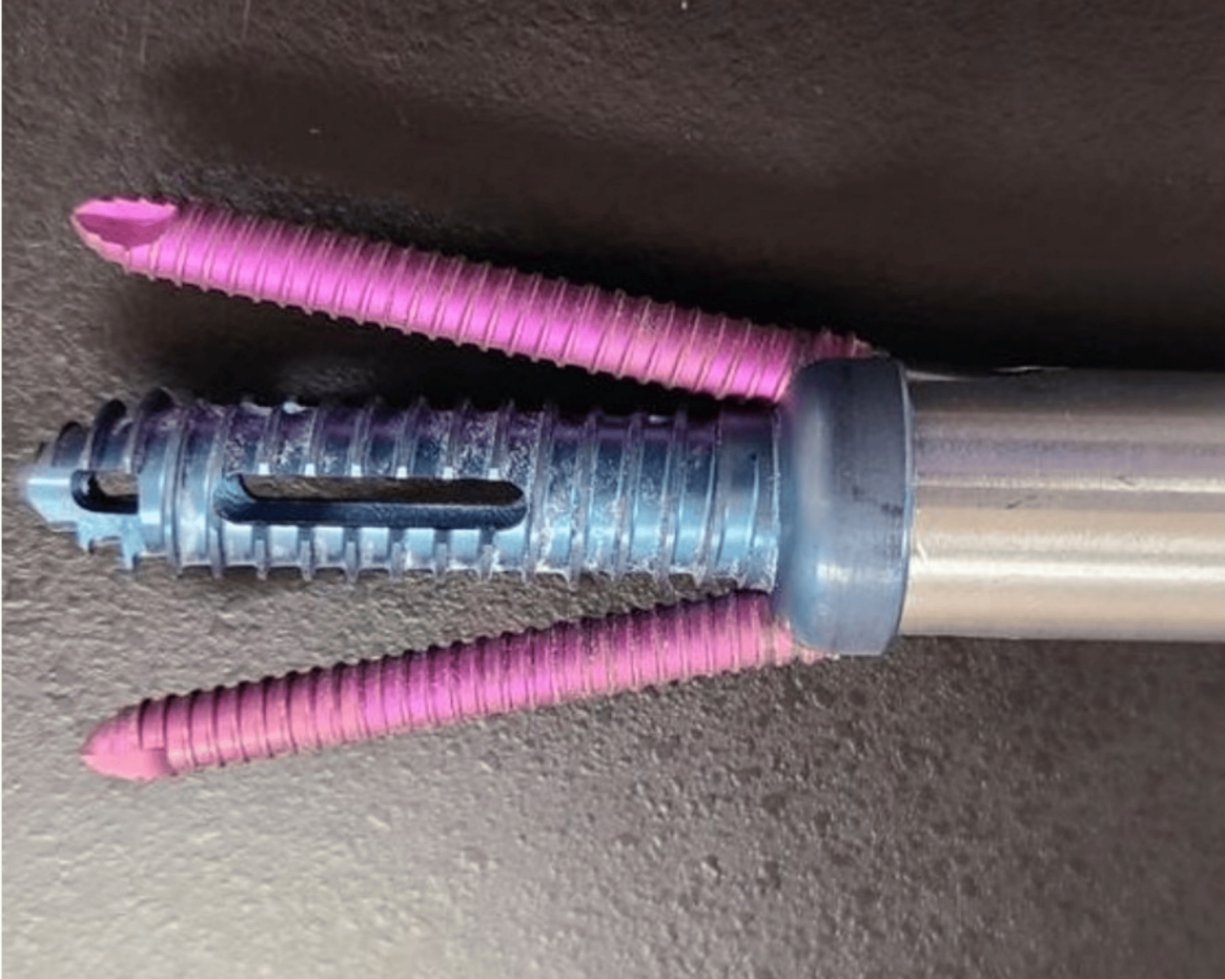
The main body of the Trident™ Screw System (in cobalt blue), as well as the superior/inferior engaging, self retaining, and side screws (in fuschia pink).

The introducer sleeve was removed from the Trident Ø13 mm main screw by turning it counterclockwise and pulling it out. Typical closure and suture procedures were performed to close the surgical site. The results of the procedure can be seen in Figure 7, along with an example screw set in Figure 8.

The screw delivery and fixation process described uptil now is performed only once and, as such, reduces the risks of vascular or neurologic injury that can be seen in other systems that require repeat surgical entry, though are still present. No patients in this study suffered any such sequela.

Before and after surgery the Oswestry Disability Index (ODI) was used as a marker of function and impairment. Although some studies have suggested that categories in the ODI can be accounted for by lowering the denominator by 5 when calculating, for the sake of this study only complete ODIs were utilized [12]. Historically, many studies on SIJ outcomes have used the Visual Analogue Scale (VAS) to report their improvement, but this metric often reports higher improvement than ODIs, which have been shown to be more sensitive [13]. In the interest of accurately detailing improvement and removing concerns for overstating results, we have chosen to omit VAS reporting.

In regards to data, ODIs close to, but still before, the surgery date were compared to an ODI obtained no less than six months after surgery. In cases where a patient underwent multiple SIJ fusions, the second ODI would be at least six months after their second surgery. The analysis of the collected ODIs was performed by a study analyst who accessed the information through a password-protected web-based database and electronic medical record system.

The statistical analysis included the use of means, averages, paired t-tests, and independent t-tests to identify any correlations and assess the outcomes of the SIJ fusion procedures. The rigorous approach to data analysis was intended to ensure the validity and reliability of the study findings.

## Results

**Figure 9:**
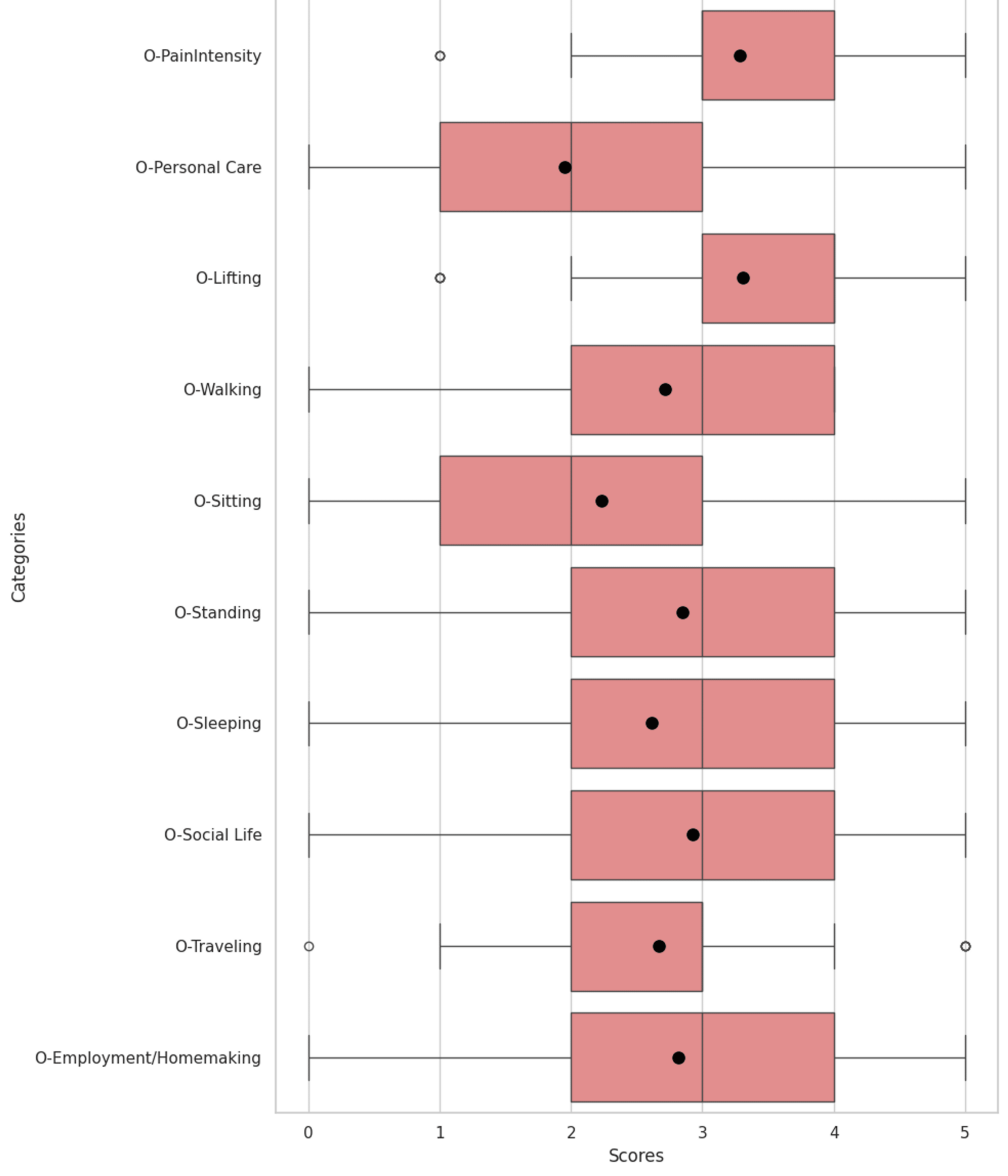
Distribution of presurgical Oswestry Disability Index categories with maximum and minimum values per category. Means are represented as dots, medians as solid lines, and outliers as open dots.

**Figure 10:**
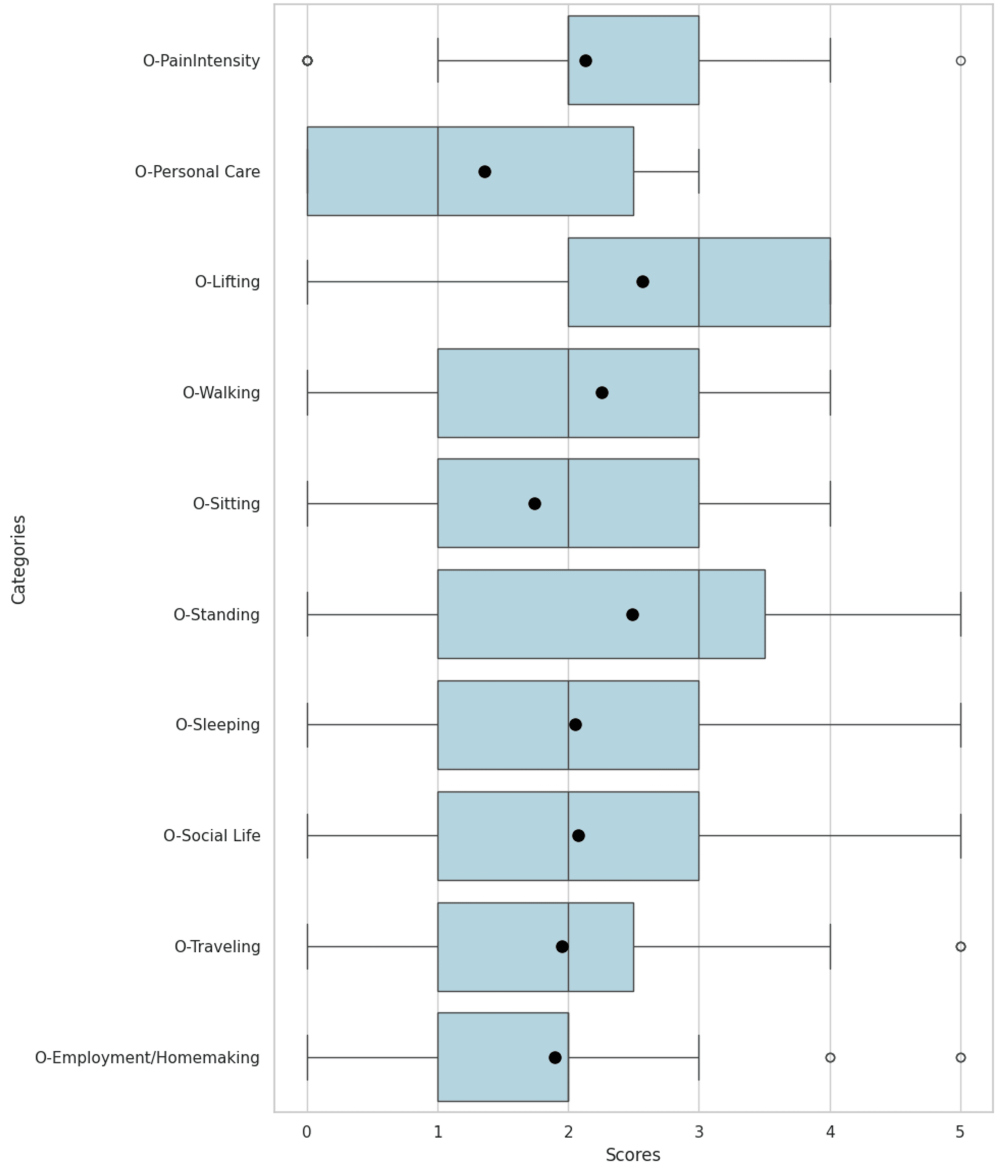
Distribution of postoperative ODI categories with maximum and minimum values per category. Means are represented as dots, medians as solid lines, and outliers as open dots.

Collected data were organized and first analyzed as a collective, comparing preoperative and postoperative values in ODIs and individual categories, as shown in Figure 9, Figure 10, and Table 1. Thresholds for statistical significance were set at p-values <0.05.

**Table 1:** Comparison of the preoperative and postoperative values for the cohort of 39 patients. The table shows t-statistics, p-values (all below the threshold of significance), and mean differences for each of the components of Oswestry Disability Index (ODI) as well as the ODIs overall. *: P-value threshold: <0.05.

Collective (39)	T-statistic	P-value	Mean difference
Pain intensity	4.760148	0.000009*	-1.153846
Personal care	2.806592	0.006357*	-0.589744
Lifting	3.340437	0.001299*	-0.743590
Walking	2.380863	0.019777*	-0.461538
Sitting	2.511977	0.014127*	-0.487179
Standing	1.830829	0.071046	-0.358974
Sleeping	2.966479	0.004025*	-0.564103
Social life	3.863751	0.000233*	-0.846154
Traveling	3.571194	0.000620*	-0.717949
Employment/Homemaking	4.558481	0.000019*	-0.923077
Overall ODI	5.885796	0.000000*	-13.692308

Statistical significance was displayed by all categories except Standing. Beyond this collective analysis, study data were further stratified into three subgroups based on surgical history. These groups were patients who underwent a single SIJ fusion and had no prior lumbar surgery, patients who underwent SIJ fusion after lumbar fusion, and patients who received bilateral SIJ fusion. These groups were analyzed independently, and statistics are presented in Tables 2-4.

**Table 2:** Comparison of the preoperative and postoperative values for the subgroup of patients who underwent a single sacroiliac joint fusion and no prior lumbar surgery. The table shows t-statistics, P-values (with Sleeping, Homemaking, and Overall categories below the threshold), and mean differences for each of the components of the Oswestry Disability Index (ODI) and the ODIs overall. *: P-value threshold: <0.05.

Unilateral sacroiliac joint fusion only (17)	T-statistic	P-value	Mean difference
Pain intensity	2.851146	0.007566*	-1.235294
Personal care	1.460593	0.153877	-0.470588
Lifting	1.767767	0.086635	-0.588235
Walking	1.191100	0.242375	-0.352941
Sitting	0.775203	0.443911	-0.235294
Standing	0.845626	0.404040	0.235294
Sleeping	2.314168	0.027234*	-0.529412
Social life	1.610954	0.117011	-0.647059
Traveling	1.223551	0.230055	-0.470588
Employment/Homemaking	3.771236	0.000663*	-1.176471
Overall ODI	2.816404	0.008250*	-10.941176

**Table 3:** Comparison of the preoperative and postoperative values for a subgroup of patients, namely, those who had undergone prior lumbar fusion. The table shows t-statistics, P-values (with Social life, Traveling, and Overall below the threshold), and mean differences for each of the components of the Oswestry Disability Index (ODI) and the ODIs overall. *: P-value threshold: <0.05.

Sacroiliac joint fusion after lumbar fusion (10)	T-statistic	P-value	Mean difference
Pain intensity	1.921538	0.070642	-0.800000
Personal care	0.768221	0.452317	-0.400000
Lifting	1.765045	0.094516	-0.600000
Walking	1.000000	0.330565	-0.400000
Sitting	1.616448	0.123388	-0.600000
Standing	1.860521	0.079230	-0.500000
Sleeping	0.536895	0.597915	-0.300000
Social life	2.371708	0.029063*	-1.000000
Traveling	2.250000	0.037195*	-0.600000
Employment/Homemaking	2.022600	0.058230	-1.000000
Overall ODI	2.647435	0.016377*	-12.400000

**Table 4:** Comparison of the preoperative and postoperative values for a subgroup, specifically bilateral sacroiliac joint fusion patients. The table shows t-statistics, P-values (with Walking and Homemaking categories above the threshold), and mean differences for each of the components of the Oswestry Disability Index (ODI) and the ODIs overall. *: P-value threshold: <0.05.

Bilateral sacroiliac joint fusion (12)	T-statistic	P-value	Mean difference
Pain intensity	3.545621	0.001814*	-1.333333
Personal care	3.187523	0.004255*	-0.916667
Lifting	2.238478	0.035633*	-1.083333
Walking	1.876166	0.073963	-0.666667
Sitting	2.137576	0.043914*	-0.750000
Standing	3.026159	0.006206*	-1.083333
Sleeping	3.079409	0.005483*	-0.833333
Social life	3.633180	0.001469*	-1.000000
Traveling	4.311172	0.000282*	-1.166667
Employment/Homemaking	1.914854	0.068603	-0.500000
Overall ODI	5.539891	0.000014*	-18.666667

**Figure 11:**
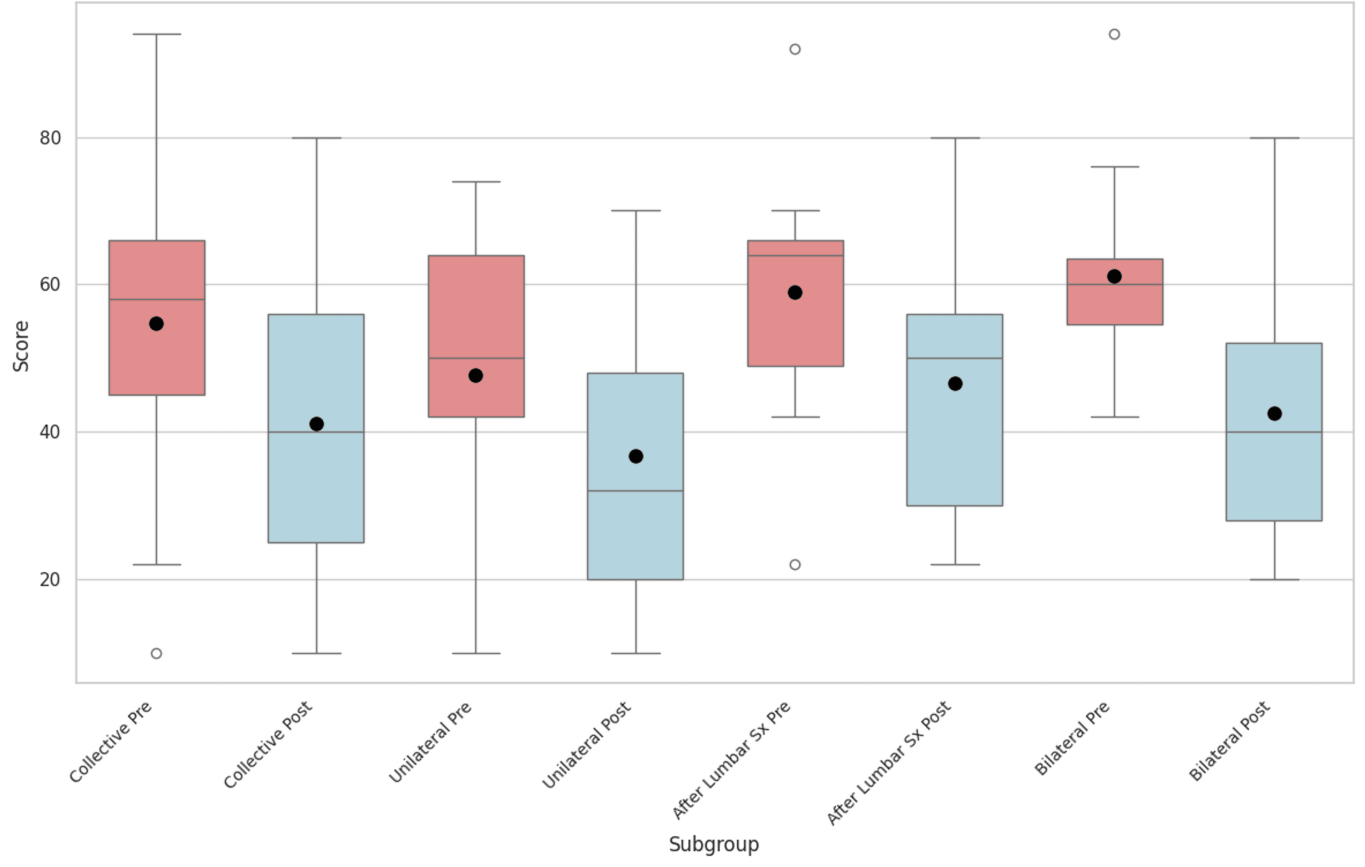
Comparisons of overall Oswestry Disability Index scores for the entire cohort as well as the assigned subgroups. The values in red correspond to preoperative scores while those in blue represent postoperative groups. For all groups, maximum and minimum scores are displayed, as well as median values (solid line in box), means (solid dot), and outliers (unfilled dots).

Many of the categories analyzed in Table 2 and Table 3 did not meet statistical significance, while Table 4 only lacks statistical significance for the categories of Walking and Employment/Homemaking. Subsequently, as a final measure, the ODIs of each subgroup and the cohort as a collective were compared in a graph shown in Figure 11. This was done to better visualize trends between subgroups and the collective.

## Discussion

The results of this study are promising as they show improvement in the average ODI by 10 points or greater, marking a clinically important difference [14,15]. Overall, 53% of patients who underwent only one-sided SIJ fusion showed improvement in clinical significance, while this rose to 70% and 75% in the subgroups of prior lumbar fusion and bilateral SIJ fusion, respectively. In addition, statistically significant improvements were also observed in a variety of ODI categories individually. When viewed collectively, there were significant improvements in all ODI categories, save for Standing, which even though showed improvement, could not be deemed statistically significant.

Even when isolated into subgroups of patients with bilateral SIJ fusion, unilateral fusion, or fusion following lumbar surgery, the overall ODIs showed statistically significant improvement. The only section that seemed to indicate a worsening of a patient’s condition was Standing in the subgroup of patients who only underwent unilateral SIJ fusion (without prior lumbar surgery), though this difference was shown not to have statistical significance. Other studies have also investigated the difference between patients who have undergone prior lumbar surgery and then underwent SIJ fusion and have found no statistical significance between the sets [15,16]. This bears relevance as prior fixations limit compensatory pressure and shock distribution; hence, in theory, one might expect additional fixation to limit necessary biomechanics and potentially lead to more discomfort.

The average starting ODIs for the study and subgroups ranged from 47.6% to 61.2%, with the unilateral group being the lowest and the bilateral group the largest. An ODI of 41% to 60% is considered a severe disability that inhibits activities of daily living, while 61% to 80% is indicative of crippling disability that affects all elements of patients’ lives, leaving them just shy of being confined to bed [17]. Both of these subgroups had average ODIs that dropped to a category of lower disability, 36.7% and 42.5%, respectively, during the six-month time frame.

Of the groups, those who had bilateral SIJ fusions showed the greatest improvement on average with a decrease in their ODI by 18.7 points. This is likely due to having a higher number of pain-generating pathologies resolved. Meanwhile, patients who had no previous lumbar surgery and underwent unilateral SIJ fusion had the lowest level of improvement in ODIs, only dropping 10.9 points, though they still graduated from severe disability to moderate disability [14]. We suspect that this can be attributed to a lower starting point of pain and relative surgical naivety. These improvements align with positive results seen with other surgical systems, such as LnK, iFuse, Rialto, and LINQ, though many of these studies encapsulate a year’s time frame and reflect a different surgical approach [18,19].

However, it is important to acknowledge the limitations of the study. The small cohort size of 39 patients, given the strict inclusion and exclusion criteria, may limit the generalizability of the results. Additionally, the study’s reliance on a single surgeon’s technique may not reflect the broader surgical community’s experience. The length of follow-up for each patient also presents a possible barrier to analysis, and future studies should aim to address this as well as the previous limitations. Despite these limitations, the study’s rigorous data analysis and the significant improvements observed in the majority of the domains provide a strong argument for the potential of this surgical technique in improving the quality of life for patients with SIJ-related pain.

## Conclusions

This study focused on clinical outcomes following a novel approach to SIJ fixation, utilizing a lateral incision to place hollow and fenestrated screws in a tri-pronged fashion across the joint, aiming to provide valuable insights into the efficacy of this innovative technique. The study demonstrated statistically significant improvements in the majority of ODI categories for patients, as well as clinical improvement. The collective data showed a positive overall trend in ODIs, with the greatest average decrease observed in patients who had bilateral SIJ fusions. Subgroup analyses further supported the efficacy of the procedure, with all groups showing clinically important differences in ODI scores postoperatively.

While the findings are promising, future studies should work on the shortcomings of this study and involve larger patient populations, a variety of surgeons to validate and potentially broaden the applicability of these findings, and, most importantly, longer follow-up periods to track patient improvements or complications. Regardless, these improvements in ODI scores post-SIJ fusion with the Trident™ Screw System suggest that this minimally invasive technique could be a valuable addition to the treatment options for chronic SIJ-related lower back pain as it has a more streamlined surgical approach and equivalent patient outcomes to other systems.

## Appendices

Appendix A

**Table 5:** Category correspondence. 1 = Pain intensity; 2 = Personal care; 3 = Lifting; 4 = Walking; 5 = Sitting; 6 = Standing; 7 = Sleeping; 8 = Social Life; 9 = Traveling; 10 = Employment/Homemaking ODI = Oswestry Disability Index

Pre-surgery date	1	2	3	4	5	6	7	8	9	10	Pre-surgery ODI	Post-surgery date	1	2	3	4	5	6	7	8	9	10	Post-surgery ODI	Overall ODI shift
6/19/2023	2	2	4	4	2	4	3	3	3	3	60	9/5/2024	2	2	3	4	3	5	2	3	1	4	58	2
7/31/2023	3	2	4	3	3	3	3	3	3	3	60	8/26/2024	0	1	1	1	1	1	1	2	1	2	22	38
5/4/2023	2	2	3	1	1	2	2	4	3	1	42	3/21/2024	0	0	3	1	1	1	1	1	1	1	20	22
5/22/2023	2	2	5	3	2	3	1	2	3	2	50	5/21/2024	2	1	1	1	0	0	2	1	1	2	22	28
4/6/2023	3	2	3	1	3	4	3	5	3	3	60	5/16/2024	2	2	4	3	1	3	1	5	1	3	50	10
11/22/2021	5	3	4	4	3	5	3	4	3	4	76	1/9/2024	2	0	2	2	3	3	3	3	3	2	46	30
1/30/2024	5	5	5	4	4	5	4	5	5	5	94	8/5/2024	4	3	4	4	4	4	4	5	5	3	80	14
12/5/2023	5	2	4	4	3	4	2	2	2	3	62	8/28/2024	2	2	3	2	1	3	1	2	2	2	40	22
10/26/2021	4	1	4	4	2	4	4	3	3	2	62	5/20/2024	3	0	1	3	1	4	3	1	2	1	38	24
10/2/2023	5	2	1	2	4	4	4	3	2	1	56	7/1/2024	3	1	2	1	2	1	2	1	1	1	30	26
9/21/2023	4	3	4	2	2	4	4	4	3	4	68	9/19/2024	3	3	3	2	3	4	4	3	3	4	64	4
4/20/2023	1	1	3	4	1	4	1	3	2	2	44	4/18/2024	2	1	4	4	1	4	0	2	0	2	40	4
1/23/2023	4	2	4	3	4	3	3	3	2	5	66	11/16/2023	3	2	4	3	2	3	2	3	2	2	52	14
7/27/2023	4	1	1	4	2	2	4	4	3	4	58	3/21/2024	3	1	1	1	2	1	1	1	1	2	28	30
7/25/2023	4	1	4	3	1	5	1	5	2	5	62	3/11/2024	3	3	4	4	1	4	5	3	2	3	64	-2
4/25/2023	3	2	2	2	1	4	2	3	2	2	46	4/1/2024	3	3	3	3	3	4	2	3	2	2	56	-10
3/6/2023	4	5	5	4	4	5	4	5	5	5	92	10/12/2023	4	3	4	4	4	4	3	5	4	5	80	12
12/16/2021	1	0	3	1	1	1	2	0	2	0	22	1/11/2024	2	0	2	1	0	2	1	1	1	1	22	0
9/19/2023	4	4	4	4	3	3	3	3	3	4	70	4/11/2024	0	0	1	2	2	3	3	1	1	2	30	40
1/9/2023	5	3	4	3	3	3	2	5	2	3	66	7/24/2023	4	3	3	3	2	2	2	3	2	0	48	18
8/15/2022	3	3	3	4	4	3	4	4	3	2	66	4/26/2023	2	3	3	3	3	3	3	2	3	3	56	10
7/10/2023	2	3	3	2	3	3	2	1	1	1	42	3/8/2024	2	2	2	2	1	1	2	1	1	1	30	12
09/22/2022	1	0	4	0	0	0	0	0	0	0	10	05/18/2023	3	0	2	3	3	3	1	0	0	0	30	-20
10/12/2023	5	3	4	2	3	3	4	4	3	2	66	07/18/2024	5	3	4	2	3	2	4	4	2	3	64	2
12/02/2021	3	3	4	2	3	3	5	4	5	5	74	06/15/2023	3	3	4	2	2	2	5	4	5	5	70	4
11/13/2023	3	0	4	4	4	4	4	3	5	4	70	06/03/2021	3	1	4	4	3	3	4	3	3	1	58	12
02/27/2023	3	2	3	4	1	2	2	2	3	3	50	12/11/2023	2	2	2	2	1	3	1	0	0	2	30	20
12/01/2022	3	1	2	1	2	3	2	2	3	4	46	11/30/2023	2	0	2	1	2	3	1	1	2	2	32	14
07/25/2023	3	1	2	3	1	3	2	3	2	3	46	03/07/2024	3	1	3	1	2	4	2	3	2	2	46	0
05/01/2023	3	3	4	3	1	1	3	3	2	3	52	01/23/2024	0	0	0	1	0	1	1	0	1	1	10	42
05/16/2022	5	1	1	2	1	1	1	1	2	1	32	12/28/2023	0	0	0	2	0	1	1	2	2	1	18	14
02/23/2023	4	2	4	3	3	3	2	5	4	4	68	04/04/2024	0	0	3	2	0	2	0	0	1	2	20	48
06/01/2023	3	1	3	3	1	1	3	2	3	5	50	04/18/2024	2	3	4	2	1	2	2	3	3	1	46	4
03/23/2023	2	1	1	0	1	0	3	0	2	1	22	12/14/2023	1	0	1	0	1	1	1	1	1	1	16	6
06/30/2022	2	1	4	0	2	1	1	0	2	0	26	02/22/2024	1	0	1	0	2	1	1	0	1	0	14	12
08/20/2021	4	3	3	3	0	3	0	2	1	2	42	05/16/2022	0	0	2	2	0	2	0	2	2	1	22	20
08/16/2023	3	1	3	3	5	2	4	4	4	3	64	07/10/2024	3	2	4	4	4	4	3	4	4	2	68	-4
09/19/2022	3	0	2	4	2	1	2	3	1	3	42	02/22/2024	2	0	2	3	1	1	1	0	5	1	32	10
03/01/2023	3	2	4	3	1	2	3	2	2	3	50	01/24/2024	2	2	4	3	2	2	4	2	2	1	48	2
